# *Salmonella* spp. transmission in a vertically integrated poultry operation: Clustering and diversity analysis using phenotyping (serotyping, phage typing) and genotyping (MLVA)

**DOI:** 10.1371/journal.pone.0201031

**Published:** 2018-07-19

**Authors:** Helen Kathleen Crabb, Joanne Lee Allen, Joanne Maree Devlin, Simon Matthew Firestone, Colin Reginald Wilks, James Rudkin Gilkerson

**Affiliations:** Asia Pacific Centre for Animal Health, Melbourne Veterinary School, Faculty of Veterinary and Agricultural Sciences, University of Melbourne, Parkville, Victoria, Australia; University of Campinas, BRAZIL

## Abstract

The transmission of *Salmonella enterica* within a vertically integrated poultry operation was investigated longitudinally over an 18-month period (2013–2014). Thirty six percent of all samples collected (1503 of 4219) were positive for salmonellae with seven *Salmonella enterica* subsp. *enterica* serovars, and one *Salmonella enterica* subsp. *salamae* serovar detected. Both *Salmonella enterica* subsp. *enterica* serovars Infantis and Typhimurium were detected in all locations sampled. *Salmonella* Typhimurium was the most frequently detected serovar (63% of serotyped samples) with 8 phage types (PT) and 41 multiple-locus variable-number tandem-repeats analysis (MLVA) profiles identified. The most frequently identified phage types were PT135a and DT135. A total of 62 PT/MLVA combinations were observed. MLVA profiles 03-14-10-09-525 and 03-15-11-11-525 were the most frequently identified and 83% of the isolates shared at least one MLVA profile with an isolate from another phage type. The use of phage typing and MLVA profiling, on their own or in combination, were insufficient to understand the complexity of the epidemiological relationships between locations within this production system. Despite the high level of apparent diversity, cluster analysis was unable to differentiate the transmission pathways of all *S*. Typhimurium variants detected within the integrated enterprise. Using additional epidemiological information, the parent breeder rearing site was identified as the most likely point of introduction of two *S*. Typhimurium isolates into the production system with subsequent dissemination to the broiler flocks via the hatchery. This complexity is unable to be resolved in the absence of intensive sampling programs at all generations of the production system.

## Introduction

The epidemiology of *Salmonella enterica* transmission within a vertically integrated chicken meat operation is poorly described. The risk factors associated with *Salmonella* infection or contamination on broiler farms and during carcase processing have been well characterized, as are putative transmission pathways within integrated broiler systems, but their mechanisms of persistence and/or spread in poultry systems are largely unknown [1]. Studies are typically limited by considering individual parts of an integrated system in isolation, and frequently limit isolate description to the *Salmonella enterica* subsp. *or Salmonella enterica* subsp. *enterica* serovar (*S*. *enterica*) with no further isolate differentiation such as phage typing [2–7]. Three Australian publications from the 1970s describe the dissemination of *S*. *enterica* serovars within integrated broiler operations [8–10]. These studies were a descriptive analysis of the occurrence of *S*. *enterica* at each component of the production system and implicated feed as the common source of introduction. There are a small number of studies that characterize *S*. *enterica* isolates beyond serovar identification across entire poultry meat production systems but none have been conducted in Australia [11–14]. Australia is unique in that *Salmonella enterica* subsp. *enterica* Enteritidis (*S*. Enteritidis) is not an endemic pathogen within the commercial poultry industry [15]. Human cases of salmonellosis attributed to *S*. Enteritidis do occur in Australia but are most frequently associated with overseas travel [16]. This offers a novel opportunity to understand *Salmonella enterica* subsp. *enterica* Typhimurium (*S*. Typhimurium) transmission in the poultry population in the absence of *S*. Enteritidis.

Understanding the relationships between the various *S*. *enterica* isolates that are transmitted between generations of birds within a vertically integrated poultry production system, beyond serovar identification, is important to quantify the effectiveness of specific control strategies at critical points in the production system. This is especially important when there are a limited number of dominant *S*. *enterica* serovars, as differentiation between isolates of the same serovar is critical for understanding the transmission epidemiology within complex production systems. When strict biosecure separation exists between generations (single source of day old chicks and eggs supplied for broiler production from this parent generation) the only route by which *S*. *enterica* can be introduced into the system via the importation of live birds is at the parent generation. Entry points for *S*. *enterica* via feed exist at both the parent and/or broiler flocks [17]. Further characterization of isolates is required to better understand points of introduction, subsequent dissemination and the effectiveness of controls within a poultry organization.

The key aims of this study were to 1. Identify the points of introduction of *Salmonella* spp. within an integrated chicken meat enterprise, and 2. To investigate introduction points and transmission pathways of any detected S. Typhimurium isolates using phage typing and MLVA profiling for isolate differentiation. To this end we conducted longitudinal sampling of key introduction (parent breeding or broiler flocks) or amplification locations (hatchery) along putative transmission paths (live birds and product; eggs) within the enterprise in order to differentiate transmission paths from entry points.

## Materials and methods

### Study population

**Fig 1:**
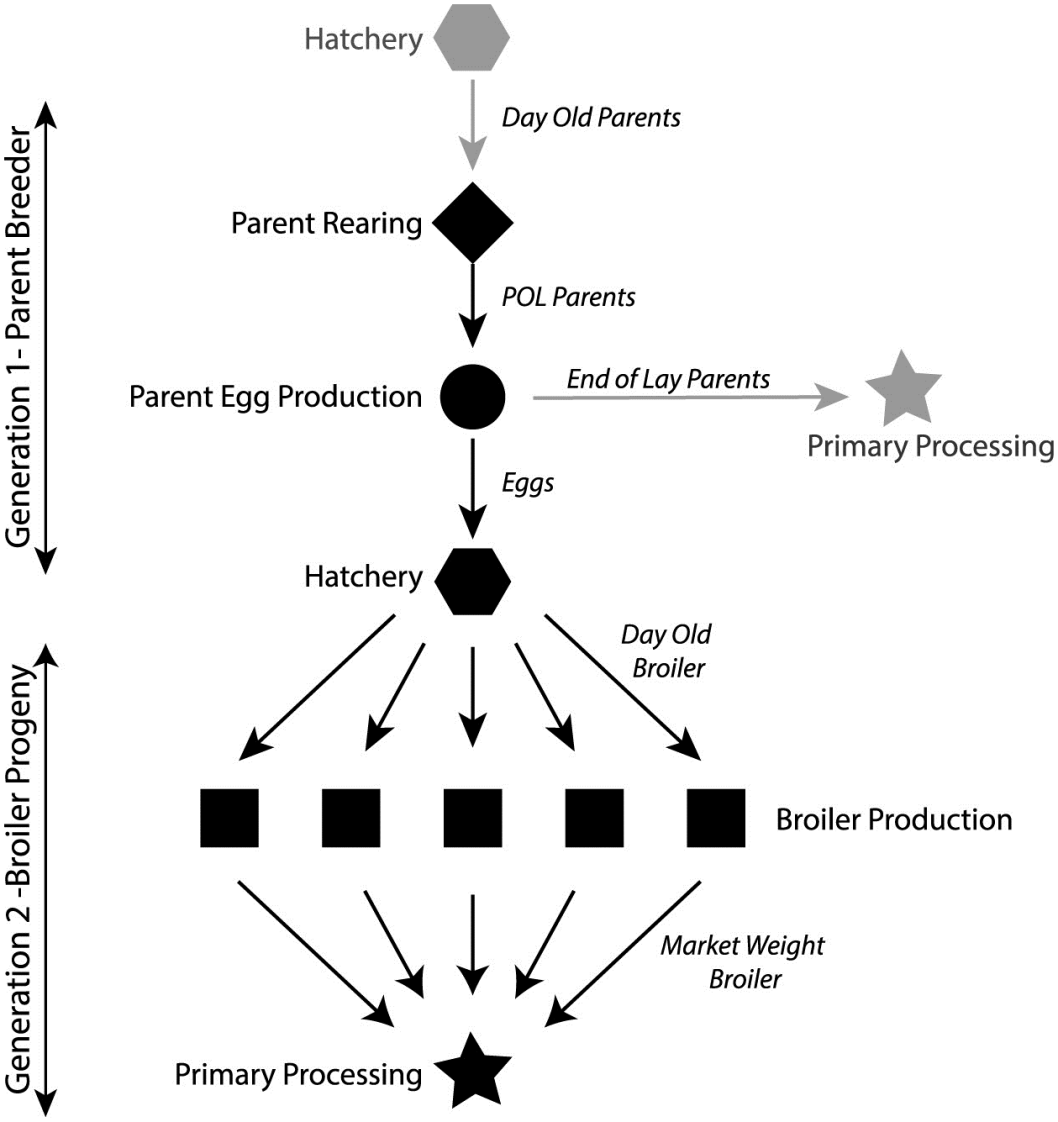
Schematic of a vertically integrated chicken meat enterprise. Solid shapes (black) represent a production site and sampling location. Bird or product (egg) transmission pathways within the enterprise are indicated by black lines between each location. Locations and paths indicated in grey represent locations or transmission pathways external to the enterprise that were not sampled. Generation 1 –Parent breeders are supplied as day old chicks from an external primary breeding company. Chicks are reared to the point of lay (POL) and then transferred to parent egg production facilities. At the onset of lay fertile hatching eggs are supplied to the hatchery for incubation. At the end of lay parent breeder flocks are depopulated and may be processed at an external primary processing plant. Generation 2 –After 21 days incubation broilers are hatched and transferred at day old to broiler production sites and reared to market weight. At market weight broilers are transported for primary processing.

This study was conducted within a single vertically integrated poultry operation. A full description of the integration and the complexity of poultry and commodity movements within the system is published elsewhere [17]. Briefly, the enterprise operates with separate sites for parent breeder rearing and egg production, the hatchery, broiler production and primary processing (Fig 1). Two generations of birds are managed within this vertically integrated poultry operation. Generation 1 comprises parent breeding flocks (roosters and hens) that produce fertile eggs for the production of Generation 2; broiler progeny. All parent generation birds are sourced from an external supplier of primary breeding stock, as day-old chicks. Day-old chicks are reared at the parent rearing sites and transferred to parent egg production facilities at the point of lay (~20 weeks of age). Parent breeders produce fertile eggs from ~23 weeks of age until 65 weeks of age. At the end of fertile egg production parent breeders are depopulated. Fertile hatching eggs are transported to the hatchery daily where they are incubated for 21 days and the broiler progeny is hatched. Day-old broilers are transported to broiler production farms for rearing (grow-out) until market weight. At market weight they are transported for primary processing. All sites are operated under strict biosecurity with no sharing or movements of equipment or people between production types within the enterprise. The only commodities entering the system during the study period were day-old parent breeder chicks and feed [17].

This study was conducted under normal farming operating conditions to evaluate routinely conducted sampling procedures for surveillance purposes. All environmental sampling was conducted as part of routine production standard operating procedures in accordance with standard industry procedures and guidelines [18–21]. As all samples were collected as part of routine veterinary care and agricultural practice this study did not require ethics approval [22].

### Study design

A prospective cohort study was conducted over an eighteen-month period between January 2013 and July 2014. The study was designed to identify *S*. *enterica* infected flocks and follow putative transmission paths for the transmission of salmonellae within the enterprise. A stratified longitudinal sampling approach was taken, to ensure samples were collected from each generation of the enterprise and from all locations identified in the previous study [17] as important points of introduction (parent breeder rearing or fertile egg production, broiler production) or amplification and dissemination (hatchery or broiler production).

**Fig 2:**
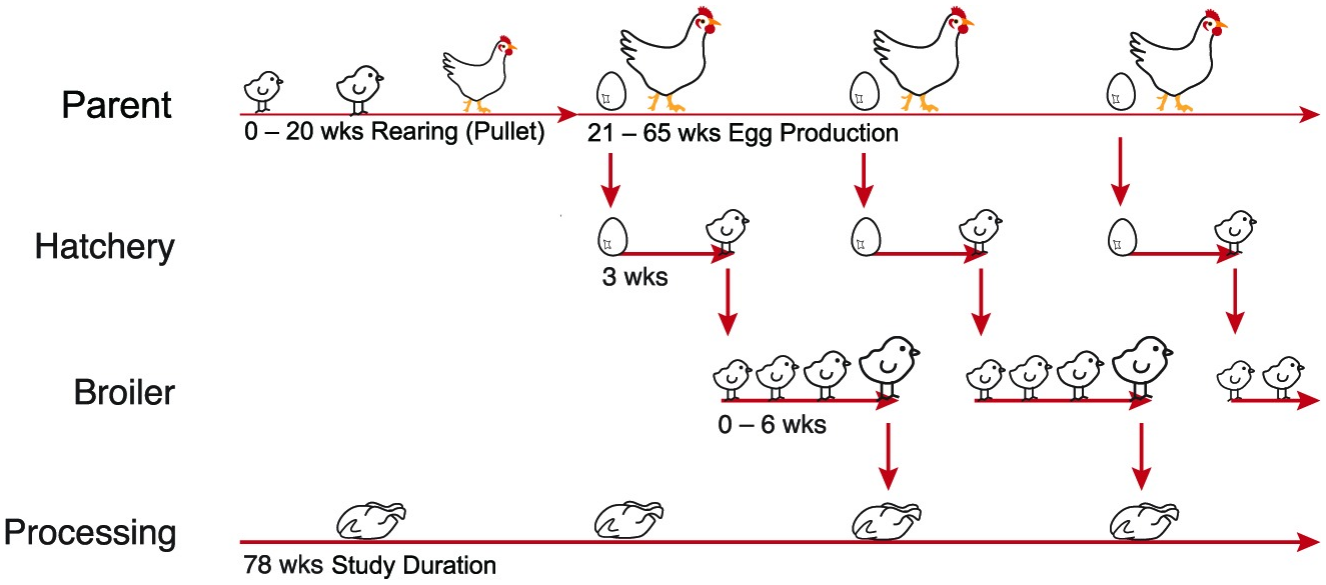
Study design schematic. Sampling occurred longitudinally within each of the two generations of the integrated system. Four *S*. Typhimurium positive parent flocks that were identified at rearing were intensively sampled (3–4 weekly) for their entire production period (65 wks). Products (eggs) or progeny derived from these parent flocks were sampled by following the putative transmission pathways from one generation to the next via the vertical paths as indicated by the red arrows. Samples were collected from day old broilers at the hatchery and 69 broiler flocks were sampled on three occasions during the grow-out period. Samples were obtained monthly (when available) from primary processing for the whole study period (75 weeks).

An intensive sampling strategy was designed, as described below, for all additional sampling. All parent breeder flocks placed as day old chicks (n = 44) were sampled at least five times during rearing. All parent breeder flocks (n = 22) in egg production were sampled at least twice during the study. Four parent breeder flocks (A-D), which were identified as positive for S. Typhimurium at rearing were recruited for intensive longitudinal sampling and were sampled 3-weekly during housing at the parent egg production facilities (20–63 weeks; 14–16 sampling events). Every three weeks (the same week as sampling was conducted at the parent egg production sites +21 days for incubation) samples from progeny of parent breeder flocks (A-D), were collected from the hatchery. For a 16 week period (Jan–May) hatchery samples from progeny of all parent breeder flocks were collected. For the same 16 week period 69 broiler chick placements were identified and longitudinally sampled at the broiler production sites during the grow-out period. Each broiler flock was sampled four times. All sheds were sampled post cleaning prior to flock placement. Day-old broiler chick samples were collected at the hatchery, and environmental samples were collected at weeks one and four and immediately after flock depopulation (Fig 2). In addition, putative *S*. *enterica* positive samples were supplied monthly from the processing plant for the duration of the study, to compare any isolates with those obtained at other points in the production cycle.

#### Sample size calculations

Sample size was calculated for a design prevalence of 1 *Salmonella* positive unit per 100 units at risk (1. Birds or 2. Environmental sites) with 95% confidence that the estimated prevalence was within 5% of the true prevalence in the unit to be tested, assuming an imperfect test. The number of birds to be sampled to demonstrate freedom from disease was estimated [23] and the number environmental sites to sample was calculated by estimating the true prevalence assuming an imperfect test [24, 25]. Test sensitivity (0.88–0.98) and specificity (0.99–1.00) were used from published laboratory testing using the same microbiological methodology [26–28]. At the lowest test sensitivity the number of birds to sample, using freedom from disease calculations for this design prevalence, can only be calculated for perfect specificity (1.00). At the lowest design prevalence ~300 bird samples would be required. To account for the indirect sampling of birds by environmental sampling and possible clustering of disease within locations and to therefore maximise the representativeness of the sample within hatches or shed environments more samples were collected than calculated. Final sample sizes used for the study were: 1000 bird equivalents or 28 environmental samples per shed per sampling event.

#### Sampling methodology

Full details of the sampling methodology can be obtained from http://dx.doi.org/0.17504/protocols.io.n6zdhf6. Standard routine sampling for management purposes was maintained during the study. All samples collected were environmental only, unless otherwise described, and no feed or water was collected from any of the sampling locations as this was outside the scope of the sampling design. The total number of flocks sampled at each generation or location differ as the production cycle is continuous. Samples from the same flock collected at one location may not be able to be collected at a subsequent location or time. Sheds were sampled repeatedly, and may have housed more than one flock during the study period.

Generation One. Parent rearing. Samples were collected from 44 deliveries of day-old chicks and the flocks established with these chicks. Ten chick box liners, referred to as chick papers, were obtained from the transport boxes on the day of each chick delivery; equivalent to 1000 chicks. Four drag swabs, each sampling ¼ shed, were collected per flock on each sampling date. Drag swabs were pooled for testing in groups of two and chick papers in pools of 10 papers per sample event. In total, there were 220 sampling events in 44 flocks, comprising 44 pooled chick paper samples and 352 drag swab samples.

Parent egg production. Twenty-eight samples were systematically obtained from each shed at each sampling event. Manure belt, egg belts, and nest box surfaces were systematically sampled by swabbing each surface the length of the shed from multiple locations to ensure the full length and width of the shed was sampled. Boot swabs were worn each sampling occasion. The same sites were sampled on each sampling occasion, sampling an area of no less than 209 m^2^. All samples were processed by sample type and location, with no pooling. Each boot swab was processed as an individual sample. In total 324 boot swabs, 533 dust swabs, 500 egg belt and 500 manure belt samples were collected from 22 flocks.

Generation Two. Broilers. At the hatchery, chick papers were collected indirectly from day old broiler chicks on the day of hatch, by the collection of chick papers randomly selected from 10 hatching trays of processed chicks; equivalent to 1000 chicks. Samples were collected from the donor flock (parent flock supplying the chicks) for each progeny broiler flock subsequently established with these chicks. In total 708 chick paper samples from 16 parent flocks were collected.

Broiler flocks were sampled on farm least three times during grow-out prior to processing. Dust samples were collected by swabbing walls the length of the shed and all internal fan guards. Two pairs of boot swabs were worn while collecting samples. No less than 40 m^2^ of floor area was sampled, equivalent to 1000 birds. In total, 263 boot swabs, 136 dust, and 138 fan swabs were collected from 69 broiler flocks.

All drag swabs [29], manure belt, egg belt and dust samples were collected using 10 cm^2^ gauze swabs pre-moistened with buffered peptone water. Two pairs of boot swabs were worn during sample collection in each shed [30].

Processing. Suspect positive *S*. *enterica* isolates (n = 183) from carcase or portion rinse samples [18] collected by quality assurance staff at the primary processing plant were provided for the duration of the study.

#### *Salmonella* spp. isolation

The full details of primary sample processing and microbiological testing for *Salmonella* spp. are available at http://dx.doi.org/10.17504/protocols.io.n6pdhdn and http://dx.doi.org/10.17504/protocols.io.n6sdhee. All samples were processed on the day of collection in accordance with the Australian Standard 5013.10–2009. Horizontal method for the detection of *Salmonella* spp. (ISO 6579:2002, MOD) [31]. In brief, buffered peptone water (Oxoid) was added to each primary sample with little mixing and each suspension was statically incubated at 37°C for 18–24 hours. After incubation, three aliquots (each 33 μL, total 0.1 mL) were taken from each primary sample and inoculated onto Modified Semi–solid Rappaport Vassiliadis plates (Oxoid). Inoculated MSRV plates were incubated under aerobic conditions at 41.5 °C for up to 48 hours, and visually examined at 12, 24 and 48 hours. Plates with swarming growth, indicative of *Salmonella* spp., were sub-cultured onto Xylose–Lysine–Desoxycholate agar (Oxoid), and either Brilliant Green Agar (Oxoid) or Cystine Lactose Electrolyte Deficient agar (Oxoid). All plates were incubated for 24 hours at 37°C. Suspect *Salmonella* spp. colonies from each sample were confirmed biochemically in triplicate using Triple Sugar Iron agar (Oxoid) and Lysine Iron agar (Oxoid), incubated for 24 hours at 37°C. All *Salmonella* isolates were confirmed positive by real time PCR in accordance with previously published methods [32]. Further biochemical testing was conducted on each isolate to differentiate S*almonella enterica* subsp. *salamae* serovar Sofia isolates. Isolates that tested positive for O–nitrophenyl–β–D–galacto–pyranoside (ONPG) (Oxoid) and able to ferment mannitol (Oxoid) were presumed to be *S*. Sofia and not submitted for typing to the reference laboratory [33].

#### *Salmonella* spp. typing

A single isolate from each sample was submitted to the Victorian Salmonella Reference Laboratory (Microbiological Diagnostic Unit) for serotyping using the Kauffman–White–Le Minor scheme [34]. Additional colonies were submitted only if more than one morpohologically distinct isolate was detected within the sample. *S*. Typhimurium isolates were phage typed using the Anderson phage typing scheme [35] and MLVA profiled. MLVA analysis was conducted in accordance with the European MLVA protocols [36] on all submitted *S*. Typhimurium positive isolates. MLVA results are presented in accordance with the Australian naming convention [37], conversion to the EU naming convention may be easily conducted [38]. The remaining positive isolates from each sample were differentiated in-house as either *S*. Typhimurium, *S*. Infantis or other *S*. *enterica* by multiplex PCR using previously published methods [32, 39, 40].

### Statistical analysis

All statistical analyses were conducted in the R statistical package unless otherwise stated [41]. Ecological diversity and cluster analysis was conducted using “vegan” [42], “BiodiversityR” [43], “FactoMineR” [44], “factoextra”[45] and “pvcluster” [46]. The distribution of phenotypes/genotypes between study locations was visualized using venn diagrams created in the “VennDiagram” package [47].

#### Multiple-locus variable-number tandem-repeats analysis (MLVA)

Two methods of MLVA analysis were implemented. 1. MLVA analysis using the goeBURST algorithm in “Phyloviz 2.0” [48–50], whereby *S*. Typhimurium MLVA relationships were visualized using minimum spanning trees (MST) of single and double locus variants. 2. Manual MLVA profile curation, whereby MLVA profiles of each phage type, with no more than two tandem repeat differences at either a single or double allele were combined into a single group [38]. This method of profile curation may be applied in public health outbreak investigations [51].

#### Differences in species composition between locations

The diversity of the species composition at each location was measured using the Shannon-Weiner index of diversity (H´). Shannon’s index (H´) is a non-parametric measure of heterogeneity combining both evenness and richness in a single measure, but is sensitive to sample size. This index accounts for both abundance and heterogeneity of the species present. The null hypothesis under investigation was that there was no difference in *S*. Typhimurium typing profiles (PT, MLVA or PT/MLVA combination) between parent, broiler and processing locations. To ensure the sample size at each location was sufficient to detect a difference between two locations with 95% confidence and 80% power the *S*. Typhimurium MLVA profile variation between and within locations was estimated. This variation was used to estimate the sample size required at each location to detect a true difference between locations using an ANOVA power calculation for multiple group comparison [52]. As more within-location (s^2^ = 21.1) than between-location (s^2^ = 2.0–3.0) variance was observed a minimum number of 50 samples per location was estimated as required to provide sufficient analytical power. *S*. Typhimurium results were aggregated by generation for all sampling events to maximize both the sample size and analytical power.

#### Dissimilarity and cluster analysis

Analysis of species composition was evaluated by measuring the ecological distance between locations. The ecological distance (dissimilarity) between sites that share the most *S*. Typhimurium typing variants is small, whereas the ecological distance between sites sharing only a few typing variants in common is large. Location dissimilarity was calculated using the Chao index [53]. The Chao dissimilarity index attempts to take into account the number of unseen species pairs that may not be detected due to the limitations of sampling where complete inventories of all “species” are potentially impossible to collect [54]. Agglomerative clustering was conducted using three methods on each distance matrix: unweighted pair group average (UPGMA), single and complete linkage. The cophenetic correlation was used to evaluate which of the three clustering methods best represented the original matrix ecological distance. Statistical significance of the correlation was estimated using bootstrapping to calculate the approximately unbiased P-value (AU) and 95% confidence intervals. If the AU P-value was greater than 95% then the clustering was considered to be supported by the data [46].

#### Principal component analysis (PCA)

The *S*. Typhimurium typing profile matrix for each site (either “location”; parent, broiler or processing, or “species”; PT, MLVA or PT/MLVA combination) was transformed to a “species profile” proportion of typing profiles (PT, MLVA or PT/MLVA combination per site) to remove the effect of extremely high or low abundance typing profiles while maintaining the original composition of the location matrices [55]. The adequacy of the normalisation of the species transformation was assessed using the Shapiro test and visualization of the qqplot [52]. Euclidean distances were calculated on the transformed species profile matrices. The ecological distance between sites was evaluated using principal component analysis. The number of significant principal components was assessed using the broken stick test. Principal components were included where the cumulative eigenvalue was greater than the equivalent broken stick value, sufficient to include no less than 75–80% of the total variance and visualized using a screeplot [52]. Typing profiles significantly contributing to the ordination were identified using the circle of equilibrium [56] and their square cosine values [57].

## Results

### Sample testing summary

Thirty six percent (1503/4219) of the samples collected were positive for *Salmonella enterica* subsp. (Table 1). The proportion of positive samples varied according to location of sample collection, with a higher proportion of *S*. *enterica* positive samples collected at the processing plant compared to other sites.

**Table 1:** Summary of all *Salmonella* testing results aggregated by sampling generation or location.

*Sampling Location*	*Parent*^1^	*Broiler*^2^	*Processing*	*Total*
No. Samples	2,769	1,267	183	4,219
No locations sampled (flocks)	38 (66)	47 (69)	1	86
No. positive samples (%)	448 (16.2)	916 (72.3)	140 (76.5)	1,503 (35.6)
*S*. *enterica* serovars	3	7	4	8
Shannon’s Index (H´)^3^	0.71	0.91	0.78	0.95
*S*. Typhimurium (%)	209 (46.7)	638 (69.7)	82 (69.2)	944 (65.3)
Phage Types	7	6	6	8
Shannon’s Index (H´)	0.74	1.43	1.29	1.22
MLVA profiles^4^	22	16	15	41
Shannon’s Index (H´)	1.52	1.96	2.39	2.26
PT/MLVA combinations	27	30	19	62
Shannon’s Index (H´)	1.85	2.67	2.61	2.75

^1^Parent breeder rearing and egg production site results aggregated,

^2^Hatchery and broiler location results aggregated,

^3^Shannon’s Index (H´) Measure of species diversity accounting for both sample heterogeneity and abundance.

^4^Number of *S*. Typhimurium isolates both Phage Typed and MLVA profiled (n = 421)

### *Salmonella* spp. serotyping

Ninety six percent of the *Salmonella* isolates were serotyped (1445/1,503) and seven *Salmonella enterica* subsp. *enterica* serovars were identified: *S*. Typhimurium (944/1445 isolates, 65%), *S*. Infantis (353/1445, 24%), *S*. Senftenberg (5/1445), *S*. Agona (3/1445), *S*. Mbandaka (1/1445), *S*. Tennessee (1/1445) and *S*. Worthington (1/1445). *Salmonella enterica* subsp. *salamae* serovar Sofia (149/1445, 10%) was also identified. Five percent of samples contained more than one serovar. *S*. Typhimurium and *S*. Infantis were the only serovars detected at all locations, while *S*. Sofia was only detected at either the broiler farms or at the processing plant. Of the 7 *S*. *enterica* serovars detected in the broiler generation, 5 were detected on broiler farms after hatch and never at the hatchery. *S*. *enterica* serovar diversity (H´) was greater at the broiler location than all others (Broiler > Processing > Parent) (Table 1).

### *Salmonella* typhimurium typing

#### Phage typing

Forty five percent (421/944) of the *S*. Typhimurium samples were phage typed and MLVA typed. Eight phage types (PT) were identified with PT135a found most frequently in 60% of the samples tested. The three most frequently identified phage types PT135a (253/421, 60%), DT135 (83/421, 19.5%) and DT193 (46/421, 11%) were detected at all sampling locations. Two phage types DT9 (9/421, 2.1%) and DT141 (10/421, 2.4%) were only detected at the onset of the study at parent breeder egg production and one DT30 isolate was identified at processing on a single occasion. Phage types DT12 (5/421,1.2%) and Untypeable (15/421, 3.5%) were also identified. *S*. Typhimurium phage type diversity (H´) was greatest at the broiler locations (Broiler > Processing > Parent) (Table 1).

#### Multi-locus variable-number tandem-repeats analysis (MLVA)

**Fig 3:**
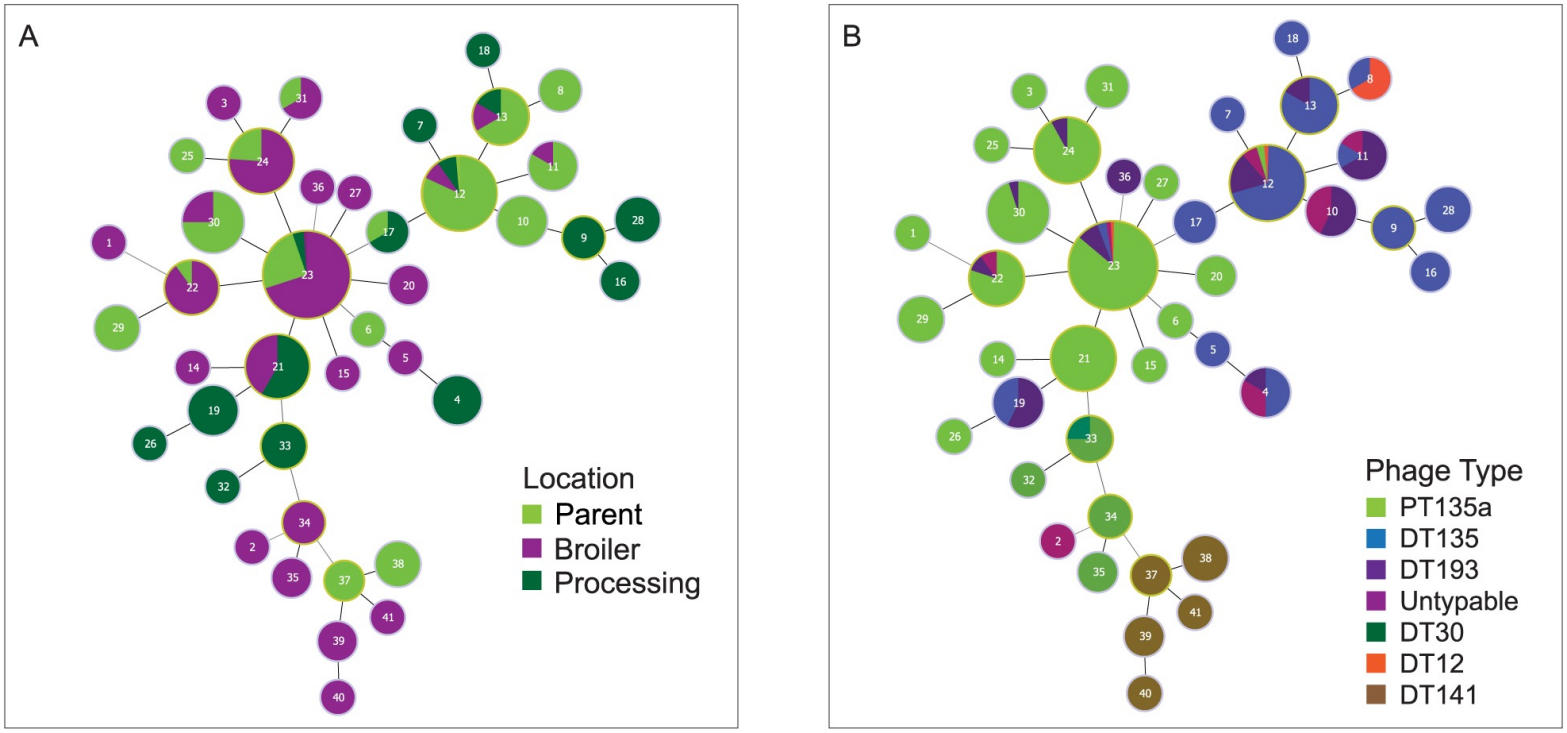
*Salmonella* Typhimurium multiple-locus variable-number tandem-repeats analysis (MLVA) minimum spanning tree (imputed using goeBURST). Each MLVA profile is represented as a single pie chart. The size of the pie chart is proportional to the number of isolates represented by that MLVA profile in the dataset and the pie segments represent either the number of locations or number of phage types associated with each MLVA profile. (A) MLVA profiles coloured by the location from where the sample was collected. (B) MLVA profiles coloured by the *S*. Typhimurium phage type associated with that profile. DT9 is not identified.

A total of 41 MLVA profiles were identified. Two MLVA profiles, 03-15-11-11-525 and 03-14-10-09-525, were detected at all locations and comprised 44% (185/421) and 14.5% (61/421) of all tested isolates respectively (Fig 3). Eighty six percent of MLVA profile 03-15-11-11-525 isolates were identified as PT135a and 70.5% of MLVA profile 03-14-10-09-525 isolates were identified as DT135. MLVA profile diversity (H´) was highest at the processing location (Processing > Broiler > Parent) (Table 1 and Fig 3A).

MLVA profile analysis identified a single minimum spanning tree (MST) combining all MLVA profiles with a single tie between MLVA profiles indicating a single locus difference between each isolate. Twenty-nine of the MLVA profiles were identified as either single (n = 14) or double locus (n = 15) variants of the two dominant MLVA profiles, indicated by a single tie from the most frequently identified profiles (MLVA 03-15-11-11-525 and 03-14-10-09-525, MLVA variants 23 and 12 respectively) in Fig 3B. MLVA double locus variant profile 03-09-04-14-525 was identified as intermediary between the two dominant MLVA profiles.

Manual curation of the MLVA profiles would reduce the number of unique MLVA profiles from 41 to 13 MLVA profile clusters containing single or double locus variants of possibly related isolates and did not alter the location that isolate groups were detected. Assuming the phage type is fixed manual curation of the isolates identified 17 PT/MLVA combinations, containing possibly genetically related isolates, and also did not change the relationships between locations.

#### Phage type/MLVA combinations

There were 62 unique PT/MLVA combinations identified from the 421 isolates. MLVA profiles were not unique to a single phage type. More than 83% of the isolates identified as DT12, DT135, PT135a and DT193 or Untypeable shared at least one MLVA profile. Phage types DT9 and DT30 isolates had a single MLVA profile in common. DT141 isolates were the only isolates to have all unique MLVA profiles, not shared with other phage types. The sharing of MLVA profiles between phage types is illustrated using the MLVA minimum spanning tree (Fig 3B).

**Fig 4:**
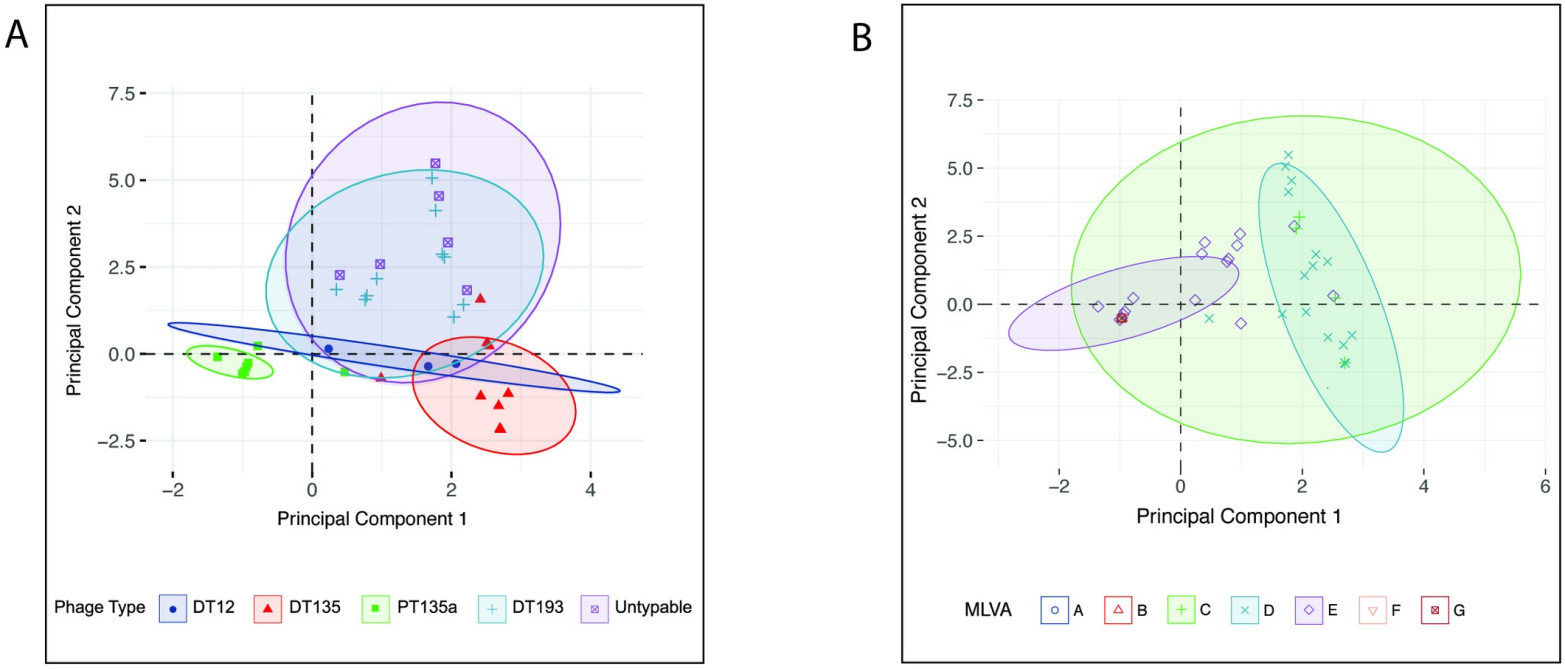
Principal component analysis of *S*. Typhimurium isolates for each typing method: Phage type and MLVA profile. The distance between each isolate is represents the Euclidean distance between isolates on the two principal component axes. Concentration ellipses illustrate isolate membership of each typing profile. Isolates are plotted at the same coordinates in both charts. (A) Ellipses identifying isolates grouped by phage type. (B) Ellipses identifying isolates grouped by MLVA profile. Only three MLVA profile groups contained sufficient members to enable ellipses to be generated (A, C, E).

The results of the principal component analysis comparing the relationships between the isolates grouped by phage type or MLVA profile are illustrated in Fig 4.

**Fig 5:**
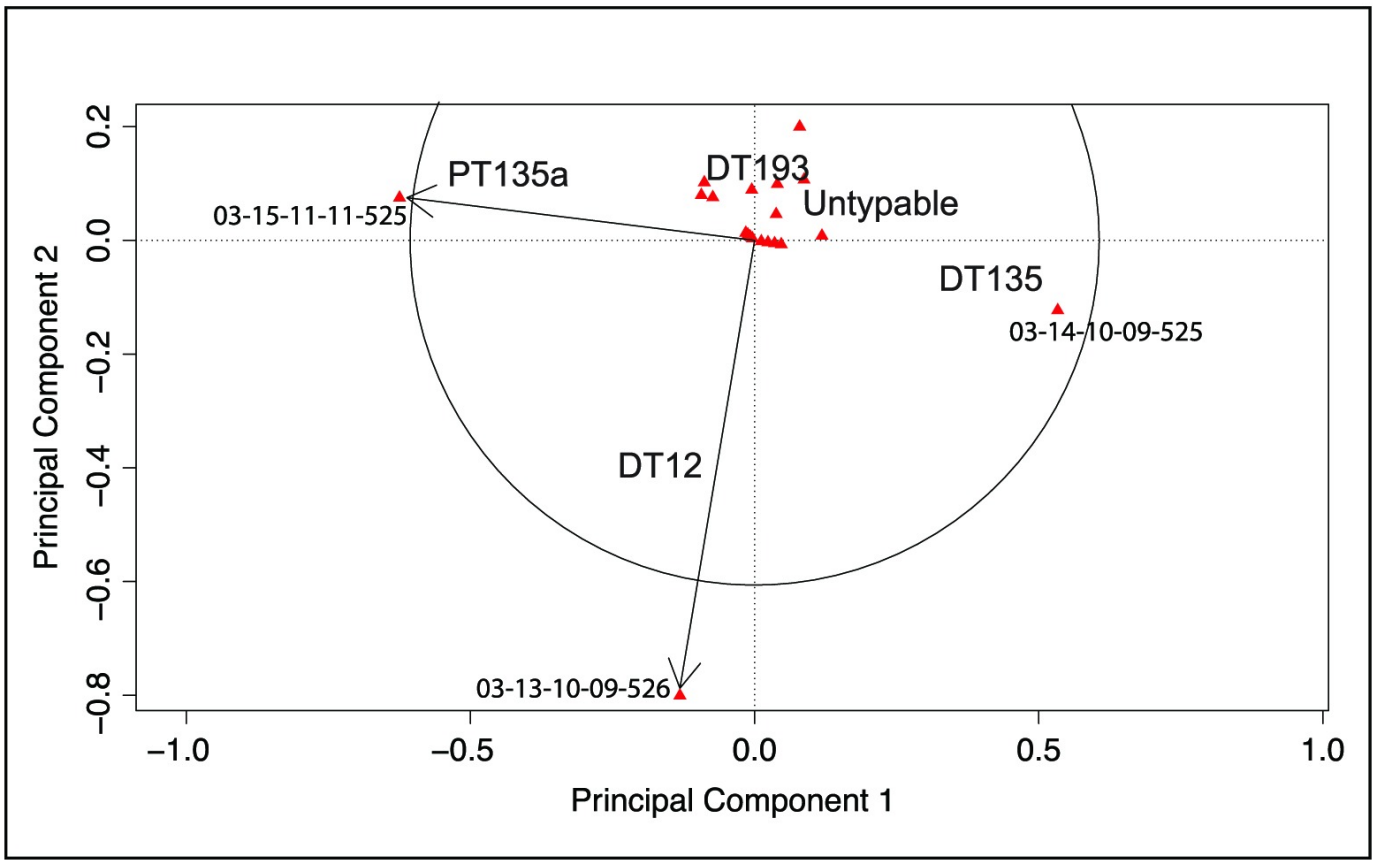
Principal component analysis of *S*. Typhimurium isolates by phage type and MLVA profile. The MLVA profile of each isolate is plotted as a point (red triangle) and the position of the phage type is identified by the phage type name. Significant MLVA profiles lie outside or close to the circle of equilibrium (black arrows). The relative distance between isolates is representative of the Euclidean distance between phage types and MLVA profiles on the two significant principal component axes.

The outlying S. Typhimurium phage types (PT9, PT141, PT30, n = 20 isolates) were removed from multivariate analysis as the number within each group was too small for accurate interpretation. The relationship between the isolates varies depending on the typing method. The relationship between the phage types is clustered (Fig 4A) but there is a high degree of uncertainty between the DT12, DT193 and Untypeable isolates, demonstrated by large overlapping confidence ellipses. There is less differentiation between the isolates when MLVA profiling is used to group the isolates, as all isolates are contained within a single confidence ellipse (Fig 4B). Using principal component analysis to compare both PT and MLVA together 88% of the variation between the two typing methods (PT and MLVA) could be explained in 2 dimensions (Table 2 and Fig 5).

**Table 2:** Phage type and MLVA variation principal component analysis versus broken stick results.

	*Principal Component*			
	1	2	3	4
Eigenvalue	0.085	0.034	0.013	0.0002
% of Variance	63	25	9	2
Cumulative % of Variance	63*	88*	98*	100*
Broken Stick % of Variance	52	27	14	6
Broken Stick cumulative %	52	79	93	100

*Component variance is greater than the broken stick variance indicating statistically significant component

Three MLVA profiles were significantly correlated with the two dimensions; 03-15-11-11-525 (ρ_component1_ = - 0.99, P = 0.001), 03-14-10-09-525 (ρ_component1_ = 0.978, P = 0.003) and 03-13-10-09-526 (ρ_component2_ = 0.95, P = 0.012) and these MLVA profiles were positively correlated (in the same quadrant) with PT135a, DT135, and DT12 respectively. The remaining MLVA patterns did not contribute significantly to the analysis, indicating they were not strongly associated with any one phage type. The methods for differentiation, phage type or MLVA profile, do not group the isolates the same, indicating substantial uncertainty in their true relationships when only these phenotypic or genotypic typing methods are considered, either alone or in combination. These typing methods give little statistical confidence in differentiating the isolates or their transmission paths within this study.

#### Clustering by location

**Fig 6:**
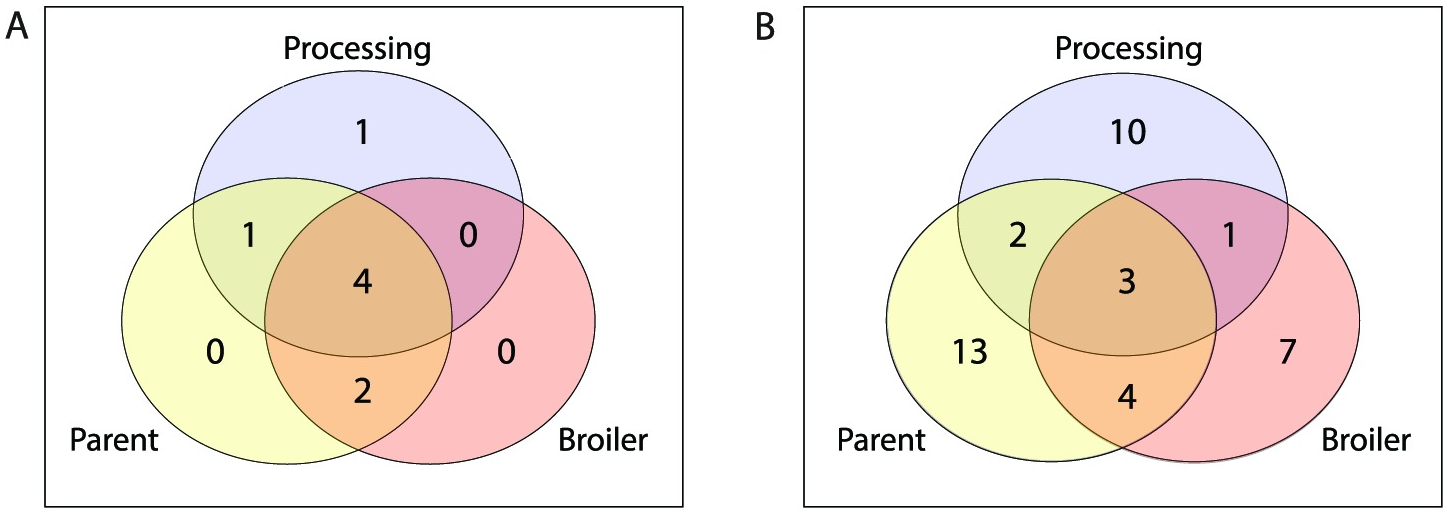
The number of unique *Salmonella* Typhimurium isolates identified by typing method detected at each location. (A) Phage Type. (B) MLVA profile.

The number of *S*. Typhimurium phage types and MLVA profiles identified at each location (parent, broiler or processing) are illustrated in Fig 6. Seven of the eight PTs were identified at the parent breeder generation and shared between the broiler generation and processing locations, A single unique PT (DT30) was identified at processing (Fig 6A). There were no PTs unique to the broilers.

**Fig 7:**
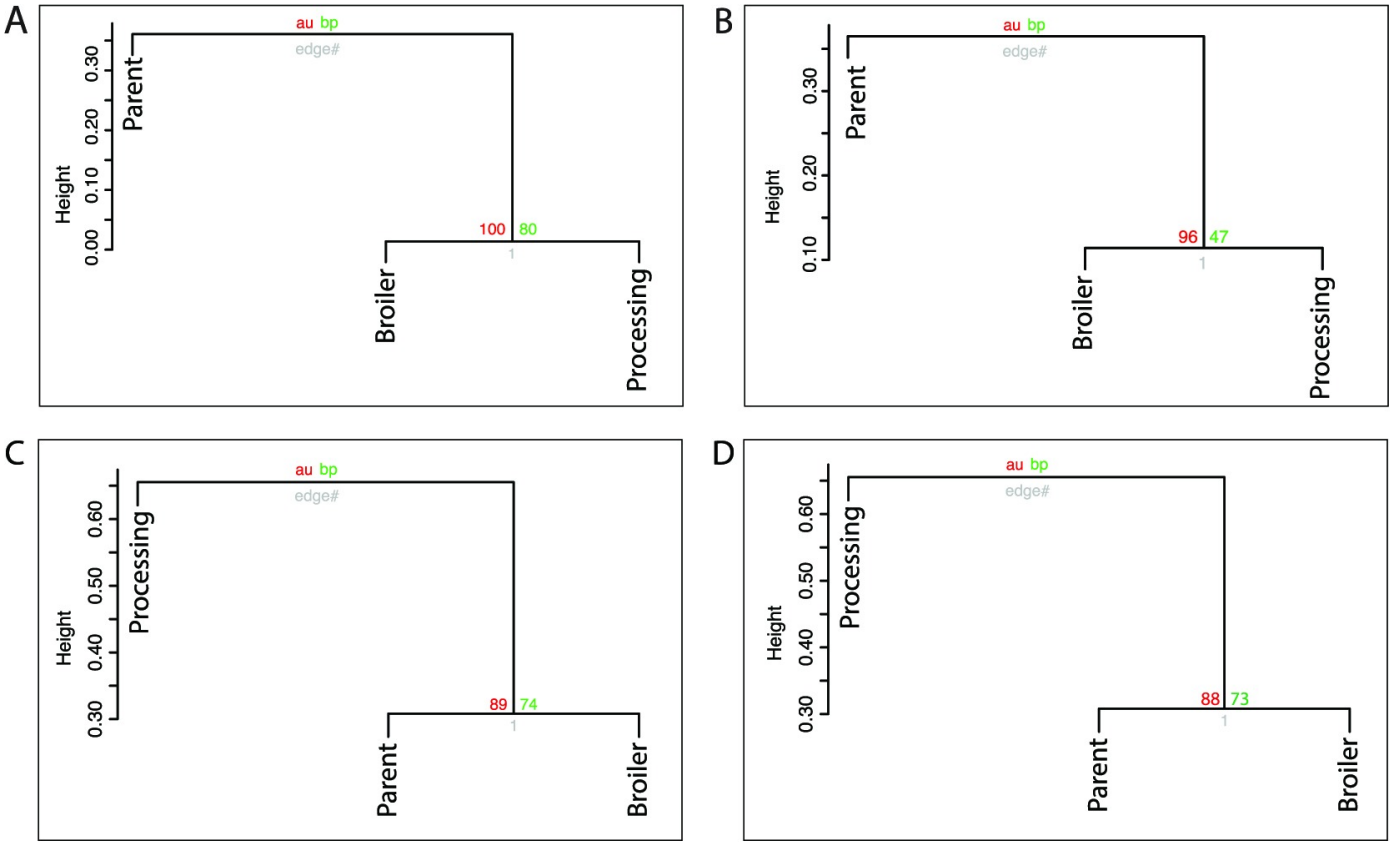
Cluster analysis of *Salmonella* Typhimurium isolates by location and typing method. Agglomerative hierarchical clustering by location (Parent, Broiler and Processing). Approximately unbiased (AU = red) and bootstrap probability (BP = green) values are indicated. A P-value less than 0.95 indicates clustering is not statistically significant. A Serotyping B Phage Type C MLVA Profile D PT/MLVA Combination.

Grouping by MLVA profile identified 10 profiles shared between locations (Fig 6B). In total 30/41 MLVA profiles were unique to any one location: parent (n = 13), broiler (n = 7) and processing (n = 10). Only 3 MLVA profiles were identified at all sampling locations. Agglomerative and hierarchical clustering demonstrated a difference between locations in the composition of *Salmonella enterica* serovars (AU P = 0.997, 95% CI [0.995, 0.999] and *S*. Typhimurium phage types (AU P = 0.959, 95% CI [0.945, 0.973]), with the broiler and processing locations clustered together, indicating they were more similar to each other than the parent location (Fig 7A and 7B). However, there was no statistically significant difference between locations when MLVA profiles or PT/MLVA combinations were used for cluster analysis; MLVA (AU P = 0.889, 95% CI [0.857, 0.921]) or PT/MLVA profile combinations (AU P = 0.893, 95% CI [0.878, 0.908]). Using these two typing methods the parent and broiler locations had more similar isolates but clustering was not significant (Table 3 and Fig 7C and 7D).

**Table 3:** Complete linkage agglomerative clustering results for each of the *Salmonella* Typhimurium typing methods.

	*Agglomerative Coefficient*	*Height*	*Cophenetic Correlation*	*P value*	*Dissimilarity (Chao)*	
					Mean (min-max)	Median
PT	0.547	0.024–0.136	0.607	0.333	0.067 (0.024–0.136)	0.040
MLVA	0.483	0.203–0.741	0.964	0.333	0.513 (0.203–0.741)	0.594
PTMLVA	0.319	0.405–0.770	0.905	0.333	0.599 (0.404–0.777)	0.618

## Discussion

In this intensive longitudinal study of a single vertically integrated poultry operation the identification of two dominant *S*. *enterica* serovars (*S*. Typhimurium and *S*. Infantis) and two *S*. Typhimurium phage types (DT135, PT135a) simultaneously across all sites was an unexpected finding. The complexity of the relationships or the transmission paths between locations and generations of birds was not able to be elucidated by further differentiation of the *S*. Typhimurium isolates by either phage typing or MLVA profiling.

Serotyping identified a greater diversity of *Salmonella enterica* serovars at the broiler generation than elsewhere (Broiler > Processing > Parent). The greater serovar diversity was acquired at the broiler farms after hatch, rather than prior. Biologically plausible sources for these isolates, given they were not found at the hatchery in day-old chicks, are feed or introduction via processing plant transportation or personnel movements.

*S*. Infantis was identified at all sampling locations, and the point of introduction was at the parent breeder egg production sites. The most likely source of this serovar at parent breeder egg production was via the feed, from which it is frequently reported [58]. There were no movements between generations (broiler -> parent) of people, vehicles or equipment, and no movement between locations (parent breeder rearing <-> parent breeder egg production) of equipment at this generation and bird movements only occur in one direction from rearing to production. People movements were very limited, under strict biosecurity arrangements including showering on and off between locations, and birds move in one direction only from rearing to egg production [17]. Parent rearing or birds were an unlikely source as *S*. Infantis was not detected at that location for the study duration. Isolates were not differentiated further so analysis of the transmission paths or points of introduction were not able to be evaluated.

*S*. Typhimurium was the most frequently detected serovar comprising 65% of all serotyped isolates. It was detected at all locations and generations of birds sampled during the study, however was only detected on two occasions at the parent breeder rearing facility. When these positive parent breeder flocks were identified, the opportunity to longitudinally sample these flocks, to determine if the *S*. Typhimurium detected at this generation was transmitted via bird or egg movements to the progeny generations and subsequently to processing, was taken. Eight phage types were identified, and 7 of these were identified at the parent breeder generation and shared with the broilers at the hatchery (Fig 4). Only 4 phage types were shared between all sampled locations, implying introductions at different locations within the enterprise. Differentiation of the isolates using MLVA profiling, identified 41 MLVA profiles and 62 PT/MLVA combinations. The apparently high level of diversity based on the MLVA profiles was unexpected and the greatest diversity was identified at processing (Processing > Broiler > Parent). Only 3 of the MLVA profiles were shared between the parent, broiler and processing locations. MLVA profiling on its own indicated the presence of a much higher number of unique *S*. Typhimurium introductions than phage typing. MLVA profiling indicated the potential introduction of at least 13 unique *S*. Typhimurium variants using manual MLVA curation and 17 when using PT/MLVA profile combinations. The variation in the MLVA profiles within six of the eight phage types, as demonstrated by PCA, confirmed that MLVA profiling did not enhance the isolate differentiation. With the exception of 3 MLVA profiles, the remaining 38 could not be correlated to a specific phage type. When using MLVA only, to identify points of introduction, the assumption the isolates are truly genetically distinct if more than 2 or three loci are unique must hold true. Similarly the PT/MLVA relationships between isolates must also be fixed, with limited scope for variation, if the assumptions for transmission are to be valid.

Agglomerative clustering is one method that can be utilised to identify if the isolates clustered between locations. The hypothesis in question was that if locations were highly correlated then they shared the same isolates, and thus shared a putative transmission path between locations. However, clustering results varied depending on the typing methodology. Only serotyping and phage typing of the isolates demonstrated significant clustering between locations (broiler and processing). Clustering between the parent and broiler generations was indicated but not statistically supported when either MLVA profiling alone or PT/MLVA combination, were used for typing. This could indicate either that there is no difference between locations, and that all the isolates are shared equally between locations, or that the typing methodology was insufficient to differentiate between clusters without a larger sample size.

In this study, MLVA profiling did not provide additional clarity and was not a useful tool for epidemiological investigation even when all complexities were known. It highlights the importance of intensive sampling within a complex epidemiological environment. The dominance of two *S*. Typhimurium PT/MLVA combinations (60% of fully typed isolates) identified at all locations and first identified at the parent rearing location supports the observation that parent sites were the most likely point of introduction of these two *S*. Typhimurium variants into the enterprise. The most likely path of transmission from parent breeder rearing to processing was via a vertical pathway between generations though the hatchery (eggs) and then dissemination to broiler and processing via movement of birds (parents -> eggs -> hatchery -> broiler -> processing). Only two potential sources for the introduction of *S*. Typhimurium into this integrated enterprise exist at the parent generation, either live birds (day-old chicks) or feed [17].

The source of the remaining *S*. Typhimurium variants at the broiler generation, either as day-old chicks at the hatchery or at processing remains unexplained. The most likely path of entry to the hatchery is via eggs but none of the variants found in the broiler birds were identified in any parent breeder flocks during the study. They may have been present at a level below detection and then amplified at the hatchery during incubation. Other than day-old chicks, feed is one possible source of *S*. Typhimurium at the broiler farms, but is rarely reported in feed [58]. The possibility of transmission of salmonellae between the processing site and broiler farms exists but pathways were unable to be clearly differentiated due to the high variability in the salmonellae as indicated by the different typing methods.

In this study multiple samples were collected and processed at each location to ensure sufficient confidence to detect *Salmonella* spp., assuming a low prevalence, and to ensure sampling was representative of the microbiological population diversity. To avoid selection bias due to sample testing methodology, multiple samples from the same environmental site, rather than multiple isolates from the same sample were typed [59–62].

Feed and water were not sampled as part of this study as the study was focused on environmental sampling. It was hypothesised that environmental sampling would detect salmonellae reported from feed if this was a primary source of introduction into the poultry flocks. *Salmonella* Typhimuirum is rarely reported in feed samples in Australian surveillance reports [63]. The results obtained from the broiler locations support the hypothesis that feed is an important source of *Salmonella enterica* serovars at this location, but it was not supported at the the parent breeder location where the diversity of the *Salmonella enterica* serovars was very low. Only two serovars were detected at parent breeders and this remained consistent throughout the study, indicating differing primary sources of introduction and transmission epidemiology within the parent (rearing and production) breeder location and the broiler production locations.

Our results highlight that, while the MLVA profiles were indicative of potential dissemination within the enterprise, they were too variable to be used without considerable curation and relatively sophisticated statistical methods to enable interpretation of their relatedness. This has been reported as a potential problem in other Australian studies [64].

These findings are comparable to other studies that demonstrate, via genotyping of *S*. *enterica* isolates, that parent breeder sites may be an important source for dissemination throughout an operation [11–13]. In contrast, studies limited to *S*. *enterica* serovar identification only, frequently implicate the hatchery and its subsequent persistent contamination as a primary source of contamination to the broiler chain but do not implicate the parent flocks as the source of contamination to the hatchery [5, 6, 65]. Differences in the supply of fertile eggs to the hatchery (such as contract versus company owned supply) may explain the subtle differences in the importance of the hatchery as a source of contamination to the broiler chain.

The limited usefulness of MLVA in the absence of other phenotyping or genotyping methods for epidemiologically complex investigations is unquestionable. Beyond confirmation of similarity in outbreak situations, under strict case definitions, the amount of variation, particularly that observed at the shortest MLVA loci (STTR-5, STTR-6, STTR-10), may be misleading over longer time periods. Substantial variation at these loci has been observed and whether this is mutation, evolution or variation due to the method of analysis (measurement error) remains to be fully explained [38, 66–68]. Further genetic characterization of both the *S*. Typhimurium and *S*. Infantis isolates by whole genome sequencing is warranted to further examine the transmission of these isolates within the enterprise.

## Conclusions

The relationships between the isolates detected in this study were revealed by intensive repeated longitudinal sampling within the same environment and the combined application and interpretation of multiple typing methods. Considered on its own, each novel MLVA profile identified could potentially misidentify the most likely transmission paths between parts of the integrated system. Without relatively sophisticated computational analysis, a substantial level of manual curation is required to utilize MLVA profiling for understanding the putative relationships between isolates. This level of curation is beyond the simple field surveillance routinely employed in most poultry production systems. The advantage in this study is that analysis has been conducted after all the results are collated; the usefulness of the typing results in isolation is limited. Extreme caution is advised when each typing method is used alone or in combination without significant sampling and epidemiological insight. To fully understand the relationships between the different isolates of the different phage types and MLVA profiles, given the substantial overlap between isolates detected in this study, further molecular characterization such as whole genome sequencing of the isolates is required.
